# Experimental Acute Pancreatitis Models: History, Current Status, and Role in Translational Research

**DOI:** 10.3389/fphys.2020.614591

**Published:** 2020-12-23

**Authors:** Xinmin Yang, Linbo Yao, Xianghui Fu, Rajarshi Mukherjee, Qing Xia, Monika A. Jakubowska, Pawel E. Ferdek, Wei Huang

**Affiliations:** ^1^Department of Integrated Traditional Chinese Medicine and Western Medicine, Sichuan Provincial Pancreatitis Center and West China-Liverpool Biomedical Research Center, West China Hospital, Sichuan University, Chengdu, China; ^2^Division of Endocrinology and Metabolism, State Key Laboratory of Biotherapy and Cancer Center, West China Hospital, Sichuan University and Collaborative Innovation Center of Biotherapy, Chengdu, China; ^3^Liverpool Pancreatitis Research Group, Liverpool University Hospitals National Health Service Foundation Trust and Institute of Systems, Molecular and Integrative Biology, University of Liverpool, Liverpool, United Kingdom; ^4^Malopolska Center of Biotechnology, Jagiellonian University, Krakow, Poland; ^5^Faculty of Biochemistry, Biophysics and Biotechnology, Jagiellonian University, Krakow, Poland

**Keywords:** acute pancreatitis, animal models, pancreatic acinar cells, pancreatic stellate cells, pancreatic ductal cells, translational research

## Abstract

Acute pancreatitis is a potentially severe inflammatory disease that may be associated with a substantial morbidity and mortality. Currently there is no specific treatment for the disease, which indicates an ongoing demand for research into its pathogenesis and development of new therapeutic strategies. Due to the unpredictable course of acute pancreatitis and relatively concealed anatomical site in the retro-peritoneum, research on the human pancreas remains challenging. As a result, for over the last 100 years studies on the pathogenesis of this disease have heavily relied on animal models. This review aims to summarize different animal models of acute pancreatitis from the past to present and discuss their main characteristics and applications. It identifies key studies that have enhanced our current understanding of the pathogenesis of acute pancreatitis and highlights the instrumental role of animal models in translational research for developing novel therapies.

## Introduction

Acute pancreatitis (AP) is an inflammatory disorder of the pancreas, which ranges from mild, self-limiting disease to a severe form that is associated with multiple organ dysfunction syndrome (MODS), high morbidity, and mortality (Hines and Pandol, 2019). Although in the last two decades advances have been made in the supportive management of AP, specific, and effective drug treatment is still lacking due to the poorly understood pathobiology of the disease (Moggia et al., 2017). Ideally, studies on the etiology, pathogenesis, and treatment of AP should be carried out on the human pancreas. However, the unpredictable nature of the disease, heterogeneity of disease presentations, and limited access to human samples, make research on human tissues impractical and often very difficult. For these reasons, experimental models have been widely used to study AP for more than a century (Gorelick and Lerch, 2017). In recent years, the most commonly used AP models are carried out on rodents (rats and mice), which are relatively inexpensive to maintain, easy to handle, accessible, and allow induction of moderate to severe pancreatic injury. These experimental models not only provide an opportunity for mechanistic studies but also enable development of therapeutic strategies.

What makes a perfect animal model? Ideally it should reflect the etiology, pathobiology, histopathology, the clinical course, and outcome of the disease in humans. However, these aims are impossible to reproduce all in one model. Our knowledge of mechanisms relevant to the human condition, the multitude of genetic and environmental factors that are likely to influence the risk of disease development and the natural history of the disease remains lacking (Gorelick and Lerch, 2017). Hence, the crucial consideration for researchers selecting a model for research studies is “What is the specific research question being asked by the study?.” Experimental AP models can be divided into *in vivo* and *in vitro* models. Further *in vivo* experimental AP models can be generally sub-divided into non-invasive and invasive models. Although these categories describe the logistic differences in inducing each respective model, certain models may have greater utility over others dependent on which mechanisms they focus on, which animal species they are induced in, and which disease outcomes are reproduced. Here we comprehensively review the history, development, and current use of important experimental AP models as well as explore their mechanisms, advantages, limitations, clinical relevance, and the scope for future work.

## Caerulein/Cholecystokinin

Early in 1895, Mouret (1895) found that excessive cholinergic stimulation causes vacuolization and necrosis of the pancreas, which are typical features of AP. Later in 1929, Villaret et al. (1929) reported the first secretagogue hyperstimulation-induced AP model by injection of acetylcholine, a cholinergic agonist, into the canine pancreas and this was later reproduced in a rat model (Leblond and Sergeyeva, 1944). Subsequently, cholecystokinin octapeptide (CCK-8) and its analog caerulein (Lampel and Kern, 1977), as well as carbamylcholine (Adler et al., 1983), anticholinesterase (Dressel et al., 1982), and scorpion toxin (Gallagher et al., 1981; Pantoja et al., 1983; Novaes et al., 1989) have been shown to induce AP.

CCK was named after its main function related to promoting contraction of gallbladder smooth muscle and bile discharge (Ivy, 1929). Later, it was found that it can act on the pancreas to stimulate the secretion of pancreatic digestive enzymes (Harper and Raper, 1943) and insulin (Kuntz et al., 2004). The fundamental mechanism of pancreatic pathology induced by CCK and its analogs is based on the action of these chemicals on CCK receptors, which in turn leads to activation of second messenger pathways related to secretion of pancreatic enzymes (e.g., amylase) in pancreatic acinar cells (PACs) like phospholipase C-inositol trisphosphate-calcium (Ca^2+^). There are also protein-protein interaction pathways that mainly regulate non-secretory processes, including biosynthesis and growth, such as three major mitogen-activated protein kinase pathways (ERK, JNK, and p38 MAPK) and several other pathways that are still unknown. While CCK-8 is most well-studied, CCK-58 is the main circulating form in humans and dogs, and the only endocrine form of CCK-58 in rats (Reeve et al., 2004). CCK-8 and CCK-58 have the same effect on Ca^2+^ signaling, zymogen activation, and cell death in PACs at high and low agonist concentrations *in vitro* (Criddle et al., 2009). A recent review has summarized the regulation of the CCK pathway in PACs in detail (Williams, 2019).

**Figure 1:**
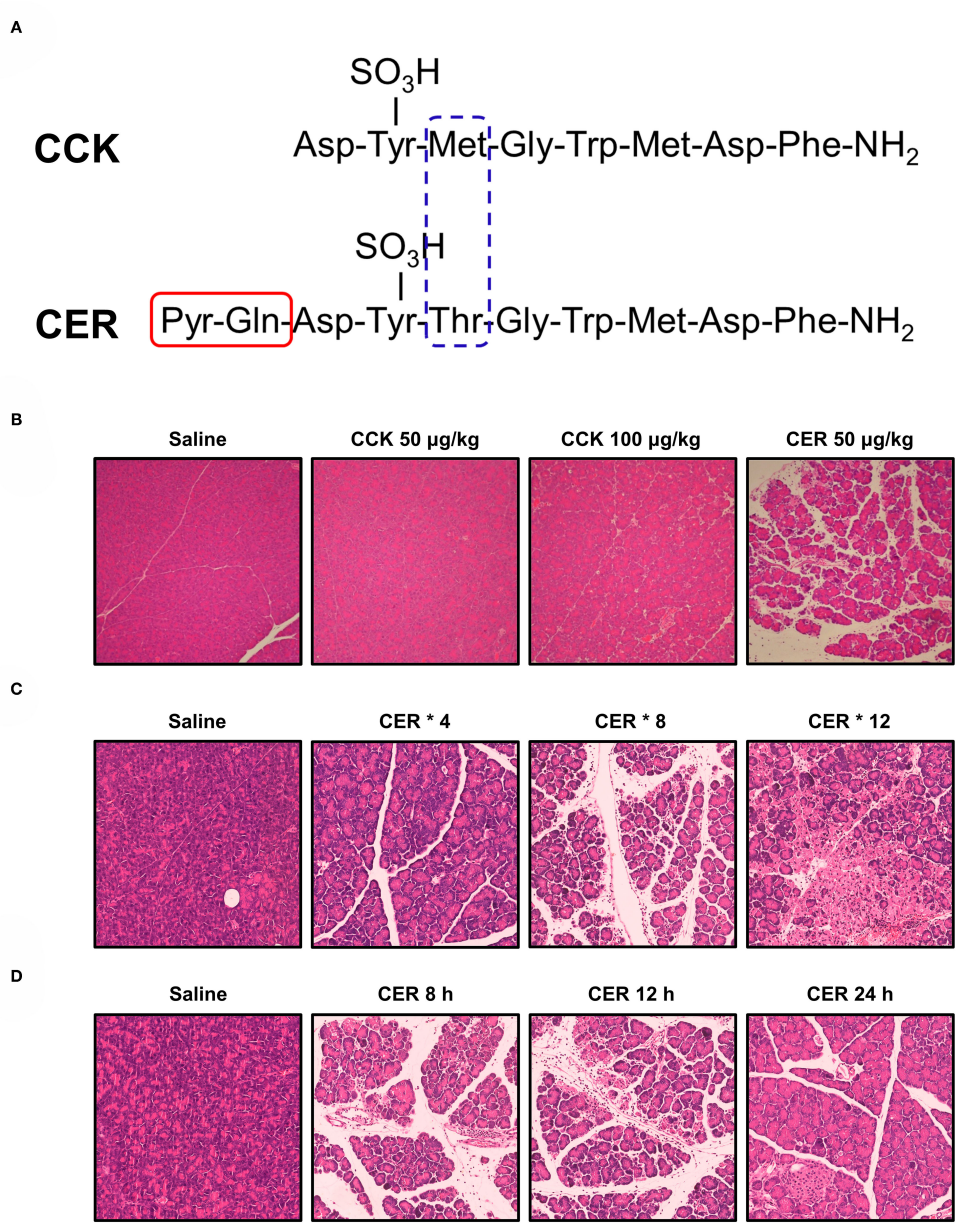
Structure of cholecystokinin and caerulein and their effects on pancreas. **(A)** Structure of cholecystokinin (CCK) and caerulein (CER). Pyr, pyroglutamic acid. **(B)** Comparison of the potency of CCK and CER in inducing acute pancreatitis (AP). Mice received seven intraperitoneal (i.p.) injections of CCK (50 or 100 μg/kg; MW: 1142.20), CER (50 μg/kg; MW: 1,352.40), or normal saline at hourly intervals. Mice were sacrificed 12 h after the first injection. While CER induced typical features of AP (edema, vacuolization, inflammatory cell infiltration, and necrosis), CCK at both doses did not cause disenable pancreas histopathological changes. **(C)** Comparison of the efficacy of different injection regimens of CER in inducing AP. Mice received i.p. injection of CER (50 μg/kg) or normal saline at 1 h apart. The mice were killed 12 h after the first injection. After 4 injections of CER, there were focally increased edema between lobules, periductal neutrophil infiltration, and minimal acinar cell necrosis; after 8 injections of CER, there were diffusely increased edema, parenchyma neutrophil infiltration, and periductal and focal acinar cell necrosis; after 12 injections of CER, there were disrupted and separated acini structure, marked parenchyma neutrophil infiltration, and focal and diffuse parenchymal necrosis. **(D)** Comparison pancreatic changes at different time after AP induced by 8 injections of CER. Mice received 8 times i.p. injection of CER (50 μg/kg) or normal saline at 1 h apart. At 8 h, there were marked pancreas histopathological changes, which peaked at 12 h and started to recover at 24 h. All experiments used C57BL/6J mice and the images were at magnification of × 200.

Caerulein, a CCK analog, was first isolated from skin extracts of the Australian green tree frog (*Litoria caerulea*) and was immediately acknowledged for its physiological activity mimicking natural hormones (Anastasi et al., 1968). CCK and caerulein have a very similar amino acid sequence, but compared to the CCK, caerulein is a decapeptide that has methionine substituted to threonine and two additional N-terminal residues (Figure 1A). Both peptides show almost the same potency *in vitro*, but caerulein is more potent to induce AP *in vivo* (Figure 1B). The increased biological activity is related to the additional N-terminal residues, and a result of the substitution of methionine for threonine (Shorrock et al., 1991). To date, caerulein remains the most widely used compound to induce AP (CER-AP) in rodents.

There is a clear dose-response relationship between the structural and biochemical changes of the pancreas in response to caerulein administration (Bieger et al., 1976b). Continuous infusion of maximal physiological doses of caerulein (0.25 μg/kg/h) causes rapid degranulation of the exocrine pancreas in rats (Bieger et al., 1976a). Administration with a supramaximal dose leads to vacuolization within the acinar cells, followed by regeneration of the pancreas (Tardini et al., 1971). At an even higher dose, caerulein causes pancreatic interstitial edema and inflammatory cell infiltration together with a significant increase of the pancreatic enzyme levels in the blood (Willemer et al., 1992). Based on the above findings, in 1977 Lampel and Kern (1977) described a non-lethal CER-AP model in rats, after which CER-AP was successfully reproduced in mice (Niederau et al., 1985).

CCK and its analogs were shown to induce pancreatic injury in a time-dependent manner in addition to a dose-dependent manner (Watanabe et al., 1984). One of the early consequences of hyperstimulation with caerulein is the formation of pancreatic oedema. This may be due to increased vascular permeability and hydrostatic pressure (Lerch et al., 1995b; Weidenbach et al., 1995). However, the exact mechanism leading to the formation of extensive oedema is still not fully understood as it does not reflect the extent of damage to PACs. This model is now widely used for the analysis of early intracellular events in AP. Although the pancreatic injury can be controlled by appropriate dosage and frequency of injections (Figure 1C) and the pancreas begins to recover after reaching its peak over time (Figure 1D), this model is generally self-limiting without MODS and lethality, which may be its biggest limitation.

To address this limitation, caerulein is often combined with other compounds to achieve increased severity of CER-AP. For example, lipopolysaccharide (LPS) in CER-AP (Sugita et al., 1997; Yamaguchi et al., 1999; Chao et al., 2006) exaggerates the inflammatory response and MODS, mimicking AP-associated sepsis. Infusion of rats with enterokinase (EK) after caerulein administration causes pancreatic necrosis, hemorrhage, and high mortality rates (Hartwig et al., 2007). Similar effects can also be replicated in mice (Hartwig et al., 2008), making it possible to undertake transgenic studies. Therefore, the “two-hit” model induced by caerulein with LPS or EK, is a useful tool to study inflammatory changes, sepsis, and bacterial translocation in AP (Xue et al., 2009). Whereas, one must remember that although the additional administration of LPS or EK results in a greater immune response in this setting, AP, at least on initiation, is primarily a sterile inflammation and this aspect of the disease is quite difficult to model.

The caerulein/CCK model exhibits the closest parallel with clinical AP induced by scorpion venom or organophosphate insecticides. It is non-invasive, easy to conduct, highly reproducible, reflects a vast number of *in vitro* studies, making it a favorable model for AP. It is also compatible with other models, sharing histopathological changes consistent with early phases of human AP (Rifai et al., 2008). All these factors explain why CER-AP is so widely accepted and commonly used by pancreatic investigators (Saluja et al., 2007; Lerch and Gorelick, 2013).

## Basic Amino Acids

Intraperitoneal (i.p.) administration of excessive doses of certain amino acids, such as L-arginine (Toma et al., 2000), L-ornithine (Rakonczay et al., 2008), L-lysine (Biczo et al., 2011b), and L-histidine (Zhang et al., 2018), causes necrotising AP in rats and/or mice. Why these particular basic amino acids induce AP has not yet been fully clarified, but is likely related to shared metabolic pathways *in vivo* determined by the structural similarities.

### L-Arginine

L-arginine-induced AP (ARG-AP) is currently the most commonly used amino acid-induced AP model in rats and mice. The effect of L-arginine on the pancreas has been extensively investigated since 1984, when single i.p. injection of L-arginine at 5 g/kg led to long lasting PAC and adipose tissue necrosis in the rat pancreas, without affecting islets and other organs (Mizunuma et al., 1984). These findings were further confirmed in the same year (Kishino and Kawamura, 1984). Higher doses (7.5 g/kg) of L-arginine can be lethal for experimental animal, whereas a dose of 2.5 g/kg only caused mild pancreatic injury. In addition, severe AP can be induced in mice by i.p. injection of L-arginine in two doses of 4 g/kg each, at 1 h apart (Dawra et al., 2007). Subsequently, in various studies either single or double injections of L-arginine were applied at different doses to induce necrotising AP in rats or mice. It is worth noting that when using the AP model induced by L-arginine, the dose, concentration, pH of L-arginine, and the strain of mice must be taken into account (Kui et al., 2015) and that D-arginine has no effect on the pancreas (Dawra et al., 2007). Severe acute inflammation of the mouse pancreas induced by L-arginine is classically followed by lung injury. There is a certain failure rate and mortality rate in this model (Hegyi et al., 2004). ARG-AP model is rarely fatal in rats, but it has a mortality rate of 5–7% in mice (Dawra and Saluja, 2012). In our laboratory, we induced AP in C57BL/6J mice (~25 g) with 4 g/kg × 2 of L-arginine, and the mortality was 1~3%. Moreover, the mortality usually occurs within a few hours after the second injection, and before AP occurs, it may be caused by metabolic disorder caused by excessive amino acids.

The mechanisms of AP induced by L-arginine remain unclear. Amino acid imbalance (Mizunuma et al., 1984), reactive oxygen species (Czako et al., 1998; Rakonczay et al., 2003), inflammatory mediators (Czako et al., 2000; Takacs et al., 2002b; Rakonczay et al., 2003), nitric oxide (Takacs et al., 2002a), cytoskeletal changes (Tashiro et al., 2001), intracellular Ca^2+^ signaling (Zhang et al., 2018), mitochondrial dysfunction (Biczo et al., 2018; Zhang et al., 2018) and endoplasmic reticulum stress (Kubisch et al., 2006) have all been postulated to be involved in this process.

### L-Ornithine

**Figure 2:**
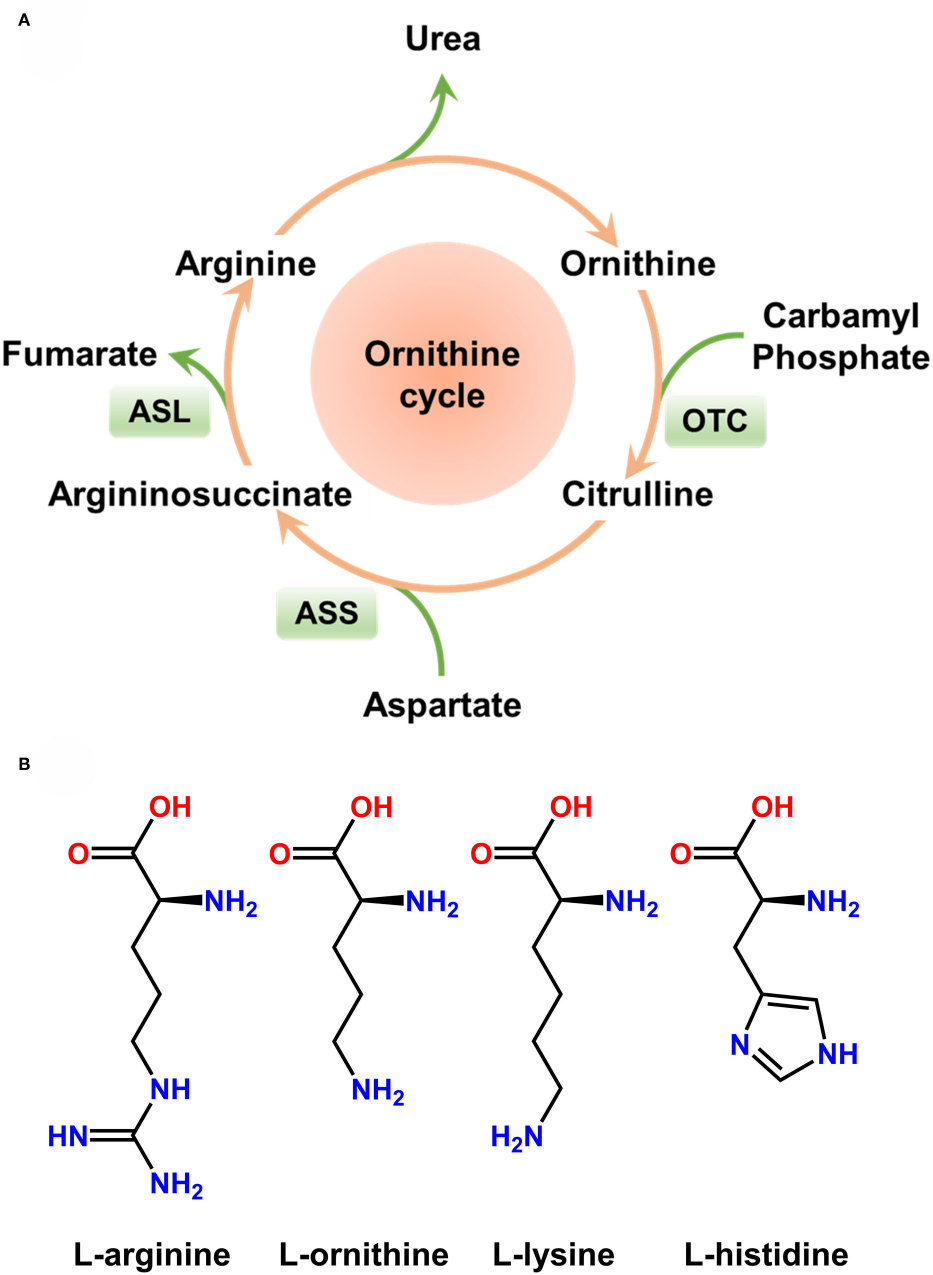
Ornithine cycle and molecular structure of basic amino acids. **(A)** Ornithine cycle. Arginine is partially metabolized into ornithine *in vivo*. **(B)** Molecular structure of basic amino acids inducing acute pancreatitis in rodents. Large doses of L-arginine, L-ornithine, L-lysine, and L-histidine have been proven to induce severe acute pancreatitis.

Rakonczay et al. reported a simple, non-invasive model of necrotising AP induced by i.p. injection of 3 g/kg L-ornithine, which is even more effective than L-arginine in rats (Rakonczay et al., 2008). Since L-ornithine is a product of L-arginine metabolism *in vivo* (in the urea cycle), it is inferred that large doses of L-arginine cause pancreatic injury at least partially through L-ornithine (Figure 2A). Biczo et al. found that pancreatic polyamine catabolism was activated in L-ornithine-induced AP, and tried to ameliorate it with metabolically stable polyamine analogs, which turned out ineffective (Biczo et al., 2010). We also tried to induce AP with L-ornithine (2 × 4 g/kg) in mice, but found the model to be excessively severe and mice were dead within few hours (Zhang et al., 2018).

### L-Lysine

*In vivo* administration of L-lysine causes early mitochondrial damage as evidenced by degenerated mitochondria shown by electron microscopy and impaired ATP synthase activity in mitochondria of isolated PACs (Biczo et al., 2011b). The mitochondrial injury appears prior to the activation of trypsinogen and nuclear factor kappa-B (NF-κB) (Biczo et al., 2011b) which suggests that the aliphatic amino acid might be more likely to cause AP due to its positive charge and L-lysine induces mitochondria injury.

### L-Histidine

Although previous studies showed that i.p. injection of 3 g/kg L-histidine had no effect on the rat pancreas (Biczo et al., 2011a), our group reported for the first time that i.p. administration of 2 × 4 g/kg L-histidine (1 h apart) to mice can induce AP (Zhang et al., 2018). L-histidine at a dose of >3 g/kg could also likely trigger AP in rats, but this has not yet been tested. One of the potential limitations is the relatively low solubility of L-histidine in physiological saline, which requires larger amounts of liquid for injections of higher concentrations of the amino acid.

The effects of different amino acids on the pancreas are summarized in Table 1. These AP models have a well-defined, gradually progressive pancreatic necrosis, and associated lung injury. Therefore, they are suitable for addressing the molecular mechanisms and regenerative processes in necrotising AP. We used L-arginine, L-ornithine, and L-histidine and found significant differences in the mechanism of pancreatic injury induced by different basic amino acids (Zhang et al., 2018). Our data indicate that the metabolism of L-arginine to L-ornithine is involved in the pathogenesis of AP induced by L-arginine (Zhang et al., 2018). It is noteworthy that basic amino acids with aliphatic side chains (Figure 2B) appear to be more effective inducers of AP. It is well-known that hereditary diseases of branched chain amino acid metabolism will greatly increase the risk of AP in human beings (Lerch et al., 2006). Therefore, the model of AP induced by amino acids may also have links to human disease. However, it must be remembered that in clinical settings human AP caused by overdose of amino acids is rare (Saka et al., 2004).

**Table 1:** Effect of different amino acids on the pancreas *in vivo*.

**Amino acids**	**Dose**	**Injection**	**Species**	**Pancreatic injury**	**References**
L-arginine	2.5 g/kg	Single	Rats	No obvious pancreatic damage	Pozsar et al., 1997; Tashiro et al., 2001; Hardman et al., 2005
	3.0 g/kg	Single	Rats	Mild pancreatic injury, e.g., focal acinar cell necrosis	Tashiro et al., 2001; Rakonczay et al., 2003
	4.0–5.0 g/kg	Single	Rats	AP with consistent moderate severity, extensive acinar cell necrosis	Shields et al., 2001; Tashiro et al., 2001; Takacs et al., 2002b; Rakonczay et al., 2003; Kubisch et al., 2006
	2.0–3.2 g/kg × 2	Twice at 1 h interval	Rats	Severe acute necrotising pancreatitis	Takacs et al., 1996, 2001, 2002b; Hegyi et al., 1997, 1999, 2000; Szabolcs et al., 2006
	3 g/kg × 2	Twice at 1 h interval	Rats	Severe acute necrotising pancreatitis	Biczo et al., 2018
	3.3 g/kg × 3	Three times at 1 h interval	Mice	Severe acute necrotising pancreatitis	Biczo et al., 2018
	4.0 g/kg × 2	Twice at 1 h interval	Mice	Severe acute necrotising pancreatitis	Dawra et al., 2007
D-arginine	4.0 g/kg × 2	Twice at 1 h interval	Mice	No obvious pancreatic damage	Dawra et al., 2007
L-alanine	4.0 g/kg × 2	Twice at 1 h interval	Mice	No obvious pancreatic damage	Dawra et al., 2007
Glycine	4.0 g/kg	Single	Mice	No obvious pancreatic damage, two doses causing high mortality	Dawra et al., 2007
L-ornithine	3.0 g/kg	Single	Rats	Severe acute necrotising pancreatitis	Rakonczay et al., 2008
	4.0 g/kg × 2	Twice at 1 h interval	Mice	All mice died within a few hours after injection	Zhang et al., 2018
L-citrulline	2.9 g/kg	Single	Rats	No obvious pancreatic damage	Rakonczay et al., 2008
L-lysine	2.0 g/kg	Single	Rats	Severe acute necrotising pancreatitis	Biczo et al., 2011b
	4.0 g/kg × 2	Twice at 1 h interval	Mice	No obvious pancreatic damage	Dawra et al., 2007
L-histidine	3.0 g/kg	Single	Rats	No obvious pancreatic damage	Biczo et al., 2011a
	4.0 g/kg × 2	Twice at 1 h interval	Mice	Severe necrotising pancreatitis	Zhang et al., 2018

## Ethanol and Free Fatty Acids

Alcohol is the second leading cause of AP worldwide (Petrov and Yadav, 2019). However, the mechanisms whereby ethanol exerts its deleterious effects are still relatively poorly understood (Clemens et al., 2016). Early animal experimental findings from acute ethanol administration via various routes have shown that alcohol increases pancreatic duct permeability, reduces pancreatic blood flow and microcirculation (Friedman et al., 1983; Foitzik et al., 1998), decreases pancreatic oxygen consumption (Foitzik et al., 1995), and induces oxidative stress (Weber et al., 1995). Whereas, ethanol alone fails to cause AP, which is in line with the human disease: since <5% alcoholics will develop clinical AP (Forsmark et al., 2016). Instead, it appears that the pancreas is sensitized to injury by alcohol consumption, and an additional factor trigger initiation of the alcohol-associated pancreatic injury. There is a dose-dependent sensitization of ethanol to CCK (or caerulein)- or bile acid-mediated PAC injury *in vitro* (Katz et al., 1996; Lu et al., 2002) and pancreatic damage *in vivo* (Foitzik et al., 1994; Andrzejewska et al., 1998; Pandol et al., 1999). A recent report showed that although alcohol feeding does not cause experimental AP, endoplasmic reticulum stress, and cell death of PACs can be triggered upon simultaneous exposure to ethanol and cigarette smoke (Lugea et al., 2017a). As a consequence, ethanol has been considered a key aggravating factor in the development of AP.

**Figure 3:**
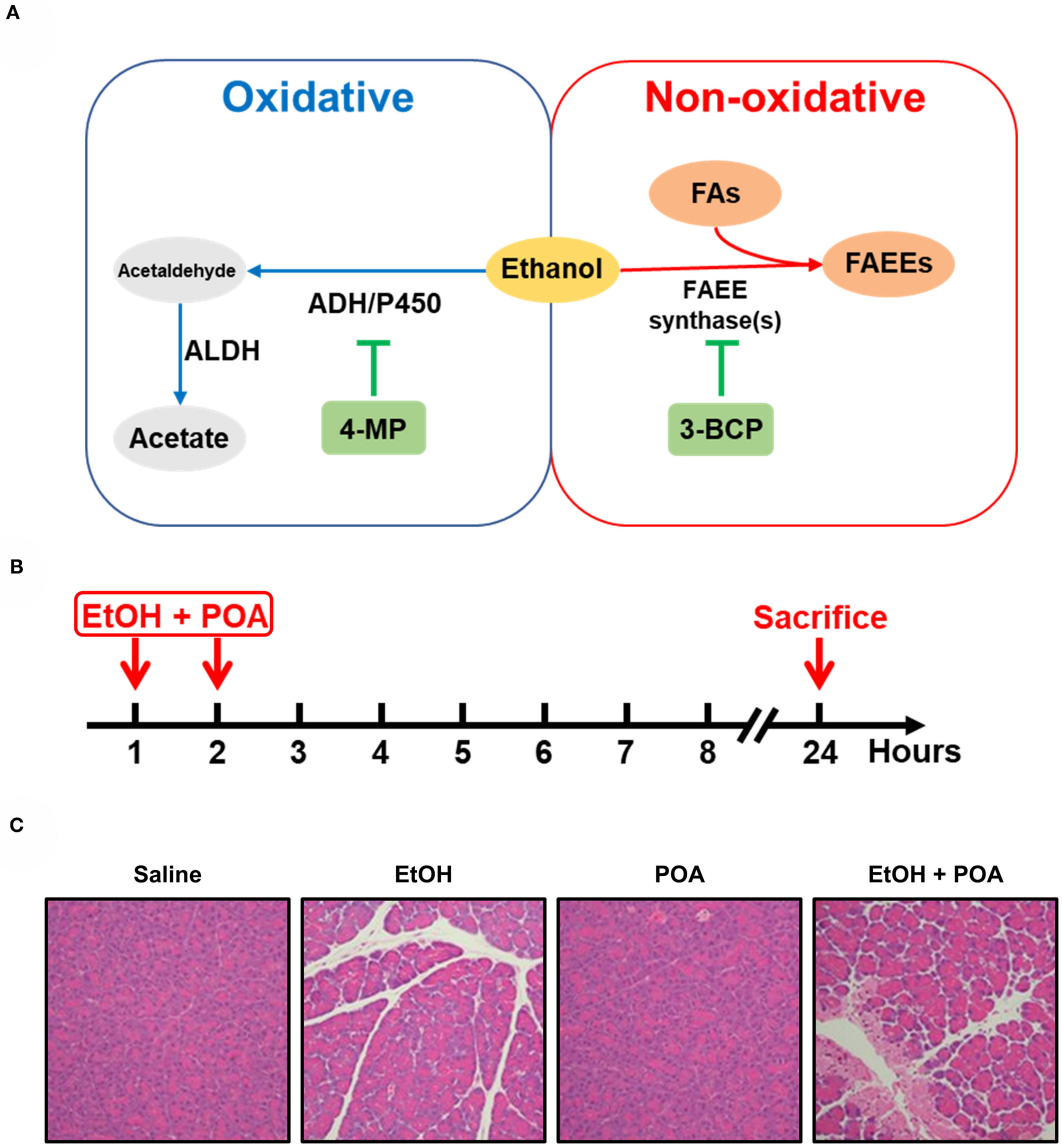
Ethanol metabolism and fatty acid ethyl ester-induced acute pancreatitis. **(A)** Non-oxidative and oxidative ethanol metabolism pathways. AHD, alcohol dehydrogenase; P450, cytochrome P450; ALDH, aldehyde dehydrogenase; 4-MP, 4-methylpyrazole; FAs, fatty acids; FAEEs, fatty acid ethyl esters; 3-BCP, 3-benzyl-6-chloro-2-pyrone. **(B)** A schematic of fatty acid ethyl ester-induced acute pancreatitis (FAEE-AP) in mice. The model was induced by concomitant administration of ethanol (1.35 g/kg) and palmitoleic acid (POA, 150 mg/kg). **(C)** Representative H&E images of pancreas histopathological slides of FAEE-AP (Huang et al., 2014).

On the other hand, ethanol metabolites by both oxidative and non-oxidative pathways (Figure 3A) have been shown to cause a number of changes that can predispose the pancreas to AP (Laposata and Lange, 1986). The oxidative metabolism of ethanol is catalyzed by alcohol dehydrogenase and cytochrome P450 2E1, resulting in the production of reactive oxygen species and acetaldehyde (Wilson and Apte, 2003; Cederbaum, 2012). Oxidative metabolism of ethanol has also been shown to sensitize pancreatic mitochondria to activate mitochondrial permeability transition pore, leading to mitochondrial failure (Shalbueva et al., 2013).

Non-oxidative ethanol metabolism is accomplished by a diverse group of enzymes known as fatty acid ethyl ester (FAEE) synthases, which combines free fatty acids (FFAs) with ethanol generating FAEEs (Wilson and Apte, 2003; Zelner et al., 2013). Alcohol consumption is associated with elevated plasma triglycerides, impaired lipolysis, and increased FFAs fluxes from adipose tissue to the liver (Klop et al., 2013). And high concentrations of FFAs are noted in the serum and pancreatic necrosis debridement fluid of patients with AP (Sztefko and Panek, 2001; Navina et al., 2011; Patel et al., 2015; Noel et al., 2016). The pancreas is particularly rich in FAEE synthases, hence the relatively high accumulation of FAEEs in this organ (Laposata and Lange, 1986; Gukovskaya et al., 2002). Direct evidence that FAEEs cause some features of AP comes from Werner et al. (2002). They found that when an ethanol infusion was combined with inhibitors of the oxidative pathway of alcohol metabolism, the injury to the pancreas was exacerbated compared to ethanol alone, and the extent of pancreatic injury was dependent on the formation of FAEEs (Werner et al., 2002). This observation fits in with the previous post-mortem findings demonstrating the presence of high concentrations of FAEEs in the pancreas of patients with acute alcohol intoxication at the time of death (Ishii et al., 1986). Direct intravenous infusion of FAEEs also induces pancreatic oedema, pancreatic trypsinogen activation, and vacuolisation in the pancreas (Werner et al., 1997). Subsequently, *in vitro* data from our group have shown that FAEEs at relatively low concentrations (10–100 μM) cause prominent cytosolic Ca^2+^ rises, leading to the impairment of the mitochondrial functions and subsequent necrosis of PACs (Criddle et al., 2006).

Based on the available literature from *in vitro* and *in vivo* studies, we postulated that a more etiologically relevant model could be developed by concomitant administration of fatty acids and non-toxic concentrations of ethanol (Huang et al., 2014). To study the effect of FAEEs on AP initiation, we i.p. injected ethanol (1.35 g/kg) and palmitoleic acid (150 mg/kg), a monounsaturated fatty acid, for two times (Figure 3B), and successfully induced pancreatic injury in mice, including marked pancreatic oedema, neutrophil infiltration, and local necrosis (Figure 3C). This model also causes thickening of alveolar membrane and infiltration of inflammatory cells in the lung, but there is no or very little effect on the liver, kidney or heart (Huang et al., 2014). Other groups reproduced this model with different FFAs, such as ethanol (1.32 g/kg) and palmitoleic acid (2 mg/kg) in mice (Vigna et al., 2014), ethanol (1.75 g/kg), and palmitic acid (750 mg/kg) in mice (Maleth et al., 2015), ethanol (1.35 g/kg), and palmitoleic acid (2 or 150 mg/kg) in hamsters (Wang et al., 2016).

The lack of a widely accepted alcohol-induced AP model in pancreatology remains a drawback in the study of alcoholic AP. The existing animal models are to date the best research tools, amongst which the Lieber-DeCarli method (Bertola et al., 2013) is the most widely used experimental model to study alcoholic diseases in rodents. The fact that AP does not occur in all patients with alcohol abuse in a clinical environment strongly suggests other factors must play a role in determining individual susceptibility. Future models based on transgenic mice harboring genetic predisposing variants combined with the administration of alcohol or its metabolites may prove to be more useful.

## Bile Acids

Although a variety of experimental AP models have been described, the clinical relevance of these models might be questioned due to the fact that they do not depend on the replication of events that are considered clinically triggering AP. One of such events is biliary reflux into the pancreas through the pancreatic duct, which is believed to be the most common cause of AP in humans (Lerch and Aghdassi, 2010).

After the pancreatic duct infusion-induced AP (PDI-AP) report by Bernard (1856), bile salts such as sodium chenodeoxycholate, sodium taurocholate (NaTC), sodium glycodeoxycholic acid, taurodeoxycholate, and taurolithocholic acid 3-sulfate (TLCS) have been used to induce PDI-AP in different species. In these models, pancreatic injury develops rapidly and is limited to the pancreatic head and body, while the pancreatic tail is much less affected. Since this model requires retrograde pancreatic duct injection, the degree of pancreatic damage is closely related to the pressure in the pancreatic duct, the type of inducer and the injection speed. Manual injection may not be steady enough, and micro-dose liquid medicine injection pump should be used instead. The frequently used PDI-AP animal models induced by bile acids in rodents are summarized in Table 2 and the recent advances have been reviewed elsewhere (Wan et al., 2012).

**Table 2:** Bile salt-induced experimental acute pancreatitis models and frequently used protocols in rodents.

**Bile salt**	**Concentration**	**Volume**	**Species**	**Effect**	**References**
NaCDC	5%	2 ml/kg	Rats	Infusion of NaCDC followed by ligation of pancreatic duct caused acute necrotising pancreatitis and associated lung injury	Sun et al., 2006, 2007
NaGDC	8.5, 17, or 34 mM	100 μl	Rats	NaGDC at concentrations of 8.5-34 mM caused progressive severe but non-lethal acute pancreatitis in rats; 17 and 34 mM NaGDC infusion produced oedematous and necrotizing pancreatitis respectively; when 200 ng EK was infused with 34 mM NaGDC, necrotising pancreatitis with systemic disturbance, and rapid lethality was produced	Terry et al., 1987; Rattner et al., 1990; Rosen and Tuchler, 1992
	5 or 10 mM	100–150 μl	Rats	Low concentration of NaGDC with i.v., caerulein 5 μg/kg/h injection for 6 h caused features of moderate onset, homogeneous moderate pancreatic injury that lasts over at least 24 h	Schmidt et al., 1992a,b
NaTDC	2, 5, or 6%	200 μl	Rats	2% NaTDC infusion caused pancreatic oedema, leukocytosis, and gradually increase of acinar cell necrosis over time until 24 h; with higher concentration at 5 or 6%, pancreatic necrosis progressed more rapidly	Jin et al., 2008, 2011; Lopez-Font et al., 2010
NaTC	3, 4.5, or 5%	1 ml/kg	Rats	Significantly increased serum amylase, lipase, and pro-inflammatory cytokine levels; pancreatic oedema, vacuolisation, inflammation, hemorrhage, acinar cell and fat necrosis; lung, liver, gastric, kidney, and brain injuries; at concentrations of 3.0, 4.5, or 5.0% induced 72 h mortality rates of 24, 71, and 100%, respectively.	Paszt et al., 2004; Yang et al., 2004; Leveau et al., 2005; Dang et al., 2007; Zhang et al., 2007; Chen et al., 2008; de Campos et al., 2008; Qian et al., 2010; Xia et al., 2010; Jung et al., 2011
	2, 4, or 5%	2 ml/kg	Mice	2% NaTC caused oedema, leukocyte infiltration, necrosis, hemorrhage, and fat necrosis of the pancreas without lung injury and lethality; higher dose of NaTC increased pulmonary BAL fluid albumin and myeloperoxidase activity, and mortality: 10 and 60% mortality rates at 24 h for 4 and 5% NaTC, respectively	Laukkarinen et al., 2007; Wittel et al., 2008
TLCS	3 mM	50 μl	Mice	TLCS 3 mM infusion caused hyperamylasemia, pancreatic oedema, inflammation, and necrosis with associated lung injury	Perides et al., 2010; Hoque et al., 2011
	5 mM	50 μl	Mice	TLCS 5 mM infusion caused hyperamylasemia, pancreatic oedema, inflammation, and necrosis with associated lung injury	Du et al., 2018
	10 mM	50 μl	Mice	TLCS 10 mM infusion caused hyperamylasemia, pancreatic oedema, inflammation, and necrosis	Michael et al., 2013; Louhimo et al., 2016

*NaCDC, sodium chenodeoxycholate; NaGDC, sodium glycodeoxycholic acid; NaTDC, taurodeoxycholate; NaTC, sodium taurocholate; TLCS, taurolithocholic acid 3-sulfate; BAL, bronchoalveolar lavage; EK, enterokinase; i.v., intravenous*.

Among these bile salts, the taurine-conjugated bile salt NaTC is the most widely used and best characterized thus far for inducing PDI-AP. The rat model of NaTC infusion provides a well-defined tool to research MODS in severe AP, mirroring the human condition. This model has high mortality rates of 24–100% (Silva-Vaz et al., 2019) and the mortality increases with the amount of NaTC injected. Similarly to the caerulein/LPS model, this model is suitable for studying bacterial translocation when combined with LPS (Yamanel et al., 2005). When the NaTC/LPS model is supplemented with trypsin, even more severe MODS is produced (Yamano et al., 1998).

A non-lethal PDI-AP model (Laukkarinen et al., 2007) produced necrotising AP in the head of the pancreas without associated lung injury. This validated mouse model (Wittel et al., 2008; Ziegler et al., 2011) allows for standardizing pancreas histopathological stimuli on many levels from the isolated mitochondria (Odinokova et al., 2009) to whole animal (Mareninova et al., 2009), especially in genetically engineered mice (Mukherjee et al., 2016). Importantly, TLCS which has been extensively characterized *in vitro*, is generally preferred over NaTC for inducing PDI-AP, especially after the identification of the G protein-coupled bile acid receptor1 (Gpbar1) present on the apical pole of PACs (Perides et al., 2010). Mice lacking this receptor (*Gpbar1*^−/−^) were completely protected against AP induced by TLCS *in vivo* as well as treatment with 500 μM TLCS of PACs isolated from these mice did not result in pathophysiological Ca^2+^ responses, intrapancreatic trypsinogen activation, and cell death that are normally seen in wild type PACs (Perides et al., 2010). Subsequently, a variety of AP signaling pathways, including intracellular Ca^2+^ overload, have been verified on this model. The severity of this model is similar to that of human diseases. Paradoxically, while this is an excellent biliary AP model, it is not an ideal AP-MODS model because it requires surgical operation.

## Pancreatic Duct Ligation

The pancreatic duct ligation AP (PDL-AP) model is an attempt to experimentally recreate the “common channel,” or the common biliopancreatic duct obstruction, postulated by Opie (1901) as the mechanistic explanation for biliary AP following a gallstone lodging at the Ampulla of Vater, causing bile reflux along the pancreatic duct. Churg and Richter (1971) first linked this model to changes in the pancreatic exocrine function. Meyerholz and Samuel (2007) demonstrated that early changes in the duct and PACs can be observed at 1–5 h after ductal ligation, and 24 and 48 h were the best time points to capture interstitial oedema and inflammatory changes of the pancreas. Therefore, several researchers use this model in rats to investigate the early stages of the disease pathogenesis (Lerch et al., 1992). Studies have shown that apoptosis is the main mechanism of cell death in the rat PDL-AP model (Walker et al., 1992).

Recent advances (Samuel et al., 2010; Yuan et al., 2011; Le et al., 2015) allow the application of PDL techniques in mice (Meyerholz et al., 2008). PDL in mice causes AP with systemic inflammation and MODS, whereas biliary duct ligation and sham surgery does not (Samuel et al., 2010). The 4-day mortality of mice in the PDL group was shown to be 100%, whereas no mortality occurred in the sham operation and biliary duct ligation groups at 15 days (Samuel et al., 2010). This model appears to mimic gallstone obstruction-induced AP with a high mortality, thus it could potentially be used for investigation of the pathogenesis of severe AP and testing new therapies. The PDL-AP model may also be suitable for studying bacterial disorders, because biliary obstruction is associated with intestinal bacterial overgrowth and translocation (Nieuwenhuijs et al., 2000). The PDL-AP model has the advantage of avoiding of the systemic application of chemical inducers and thus undesirable side effects, but it requires surgery, which can be challenging particularly in small animals.

## Endoscopy

Since its first description in the late 1960s as a diagnostic technique (McCune et al., 1968), endoscopic retrograde cholangiopancreatography (ERCP) has evolved over the years to a predominantly therapeutic tool (Cotton, 2012). Compared with other endoscopic examinations, ERCP carries a higher potential for complications (Andriulli et al., 2007). Post-ERCP pancreatitis (PEP) is one of the most frequent complications of ERCP with an incidence of 1.5–15% (Parekh et al., 2017). However, the exact etiology as to why AP develops in some patients is unknown, some risk factors are gender, age, physician experience, and previous history of PEP (Radadiya et al., 2019). There are several potential underlying mechanisms of pancreatic injury during ERCP, including mechanical, thermal, chemical, hydrostatic, enzymatic, and microbiologic insults (Parekh et al., 2017).

Early in 1979, Kivisaari et al. imitated the process of ERCP by retrograde infusion of meglumine in various concentrations for 30 s at a pressure of 20 to 50 mmHg, which proved sufficient to produce evidence of both gross and microscopic AP in rats after 4 days such as oedema, leukocytosis, necrosis, and hemorrhage, atrophy, and early fibrosis (Kivisaari, 1979). Since then, different drugs, including interleukin-10, gabexate mesylate, heparin or somatostatin, nitrate derivates or diclofenac have been tested to reduce the incidence, and severity of PEP (He et al., 2003; Hackert et al., 2004; Folch-Puy et al., 2006; Xiong et al., 2007; Haciahmetoglu et al., 2008; Jin et al., 2015). He et al. described a model of PEP based on the hypothesis that a constant, relatively high pressure of an intraductal injection should cause AP through disruption of the ductal integrity (He et al., 2003). Contributing factors to the injury may include chemical toxicity from the contrast agent, disruption of pancreatic ducts, and even a rupture of acinar lobules as a result of forceful injection of contrast material. Increased pressure within the pancreatic duct has been indirectly implicated as a cause of PEP as multiple studies have shown that placement of a pancreatic stent following ERCP in high-risk patients reduced the incidence of PEP (Freeman and Guda, 2004). The model was carried out in rats by retrograde infusion of meglumine into the common biliopancreatic duct at a high pressure (50 mmHg) thus inducing typical histopathological changes of AP with significant increases of serum amylase and pancreatic myeloperoxidase activity at 24 h (He et al., 2003; Xiong et al., 2007). The model mimicked the procedure of ERCP with meglumine manifesting as edema, inflammation, necrosis, and hemorrhage. This was consistent with clinical PEP, which usually is relatively mild. However, the role of applied pressure alone has been debated after Hackert et al. (2004) and Folch-Puy et al. (2006) suggested that the contrast itself may be playing a role in the development of PEP. Hackert's work showed the inflammatory reaction develops in the pancreatic tissue when duct ligation is combined with intraductal infusion of the contrast medium (Merriam et al., 1996; Hackert et al., 2004).

**Figure 4:**
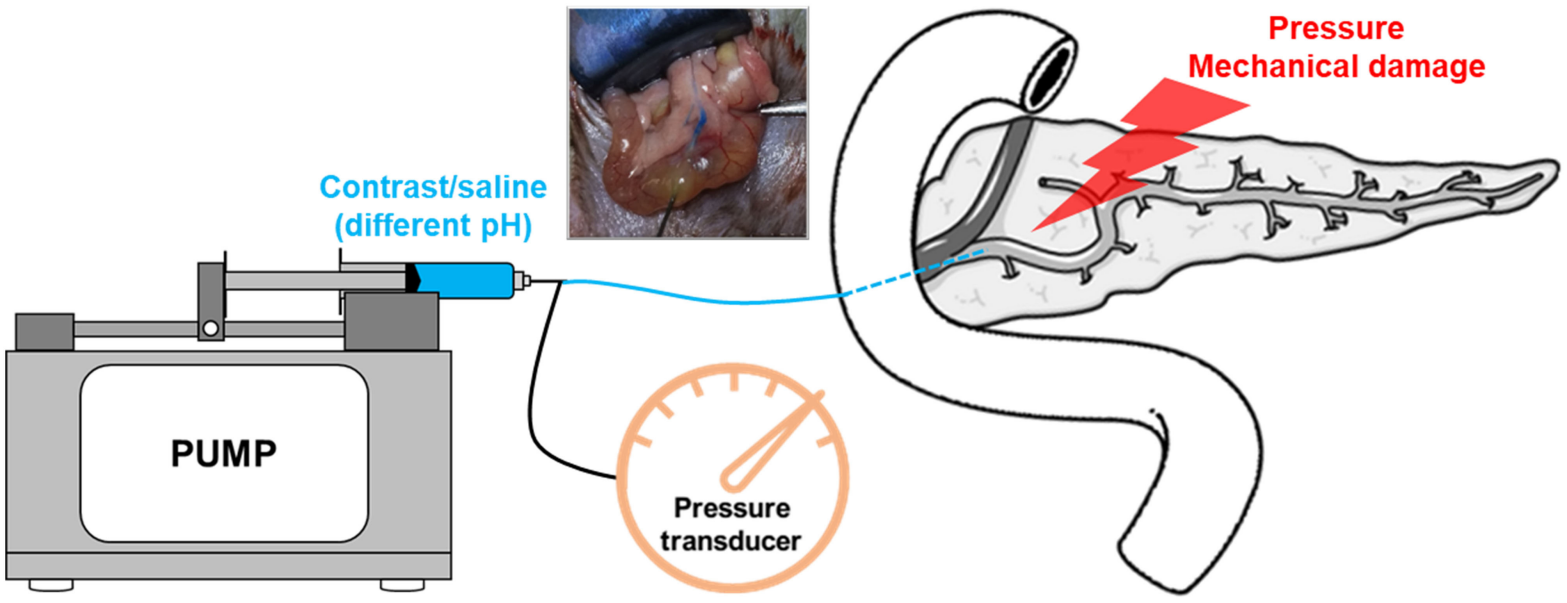
A schematic of experimental post-ERCP pancreatitis model. The model is induced by performing retrograde biliopancreatic ductal infusion, and the pump is used to control rate or pressure. Intraductal pressures can be monitored using a pressure transducer.

In addition, other studies show that intraductal regulation of pH (Noble et al., 2008) and the mechanical damage caused by direct papillary trauma (Bozkurt et al., 2013) were found to affect the onset of AP, and might be an important factor in PEP. What is more, some studies show that no correlation was detected with increasing pressure or with the use of contrast agent (Haciahmetoglu et al., 2008). Recently, Jin et al. (2015) and Wen et al. (2018) have developed a novel model of PEP in mice. Their major benefit is the ability to complement pharmacological inhibition of calcineurin along with the use of calcineurin knockouts (Wen et al., 2018, 2020). Besides, pancreatic injury after retrograde injection of contrast material has also been described in larger animals such as dogs. The frequently used PEP animal models induced by different pressure and contrast in rodents are summarized in Table 3. These models ultimately represent a combination of pancreatic ductal pressure and exposure to contrast agents, both potentiating outcomes (Figure 4).

**Table 3:** Post-ERCP pancreatitis models.

**Species**	**Method**	**Mechanisms**	**Intervention**	**Effectiveness**	**References**
Rats	Meglumine (30%, 0.4 ml) of 50 mmHg	Contrast and pressure	CP-96345 (a NK1 receptor antagonist)	Yes	He et al., 2003
Rats	Ultravist (1.2 ml/kg, 5 min) of 30 mmHg	Contrast	Heparin	Yes	Hackert et al., 2004
Rats	Low osmolarity contrast medium meglumine/sodium ioxaglate (Hexabrix 320) 10 μl/kg	Contrast	Rosiglitazone (a PPARγ agonist)	Yes	Folch-Puy et al., 2006
Rats	Meglumine (30%, 0.4 ml) of 50 mmHg	Contrast and pressure	Thalidomide	Yes	Xiong et al., 2007
Rats	Isotonic NaCl solution (0.5 ml)/diluted contrast agent (50%, 0.5 ml) of 10, 25, and 50 mmHg	Pressure and contrast	/	/	Haciahmetoglu et al., 2008
Rats	400 μl, 50 mmHg (pH = 6, pH = 6.9, or pH = 7.3)	Contrast pH	Resiniferatoxin (a TRPV1 agonist)	Yes	Noble et al., 2008
Rats	Semi-dilute non-ionic contrast agent/saline (0.5 ml) of 30 mmHg	Pressure	/	/	Bozkurt et al., 2013
Mice	Iohexol, iopamidol, or normal saline (50–100 μl) were infused at 10–20 μl per min for 5 min	Pressure and contrast	FK506 (calcineurin inhibitor), calcineurin Aβ-deficient mice	Yes	Jin et al., 2015
Mice	Saline with a constant hydrostatic pressure	Pressure	FK506 (calcineurin inhibitor), calcineurin Aβ-deficient mice	Yes	Wen et al., 2018

*NK1, neurokinin-1; PPARγ, peroxisome proliferators-activated receptor γ; TRPV1, transient receptor potential vanilloid-1*.

## Other Experimental AP Models

In recent years, pancreatic genetics has made great progress, and the identification of mutations in genes involved in the trypsin-dependent pathway is a significant milestone in understanding pancreatitis onset (Mayerle et al., 2019). Genetic experimental animal models, based on genetic techniques (transgenic, knock out, knock in, or knock down), have provided *in vivo* compelling evidence of concept of premature intrapancreatic activation of digestive proteases (Geisz and Sahin-Toth, 2018; Jancso et al., 2018; Sendler et al., 2018; Geisz et al., 2019; Gui et al., 2020; Jancso and Sahin-Toth, 2020). For example, an innovative new model has recently been developed based on the T7D23A knock-in mouse, which carries a heterozygous p.D23A mutation in mouse cationic trypsinogen (isoform T7) (Geisz and Sahin-Toth, 2018). T7D23A mice develop spontaneous AP with pancreatic edema, inflammation, and necrosis with serum amylase elevation at an early age progressing to features of chronic pancreatitis, with clinical relevance to hereditary pancreatitis (Geisz and Sahin-Toth, 2018). Using genetically manipulated mice, the mis-folding–dependent, ductal, NF-κB, and cytokine signaling pathways have also been extensively studied (Bi and Ji, 2016; Mayerle et al., 2019).

Other AP models that have been reported in the literature generally have not gained prominence due to their respective limitations. Choline-deficient, 0.5% DL-ethionine supplemented diet-induced AP was commonly used before, but it is gradually eliminated because of some limited conditions (sex, age, and non-specific pancreatic toxicity) and clinical relevance (Lombardi and Rao, 1975). The immune-induced model may represent immune factor-induced AP in humans, however, the difficulty to set it up, its limited reproducibility and other limitations have thus far hampered its use (Janigan et al., 1975; Seelig and Seelig, 1975; Nevalainen et al., 1977; Nevalainen, 1978; Jancar et al., 1988; Letko et al., 1990; Kanno et al., 1992; Axelsson et al., 2008). A haemolysis-induced model has been used to mimic AP patients under haemolysis (Saruc et al., 2003). Dibutyltin dichloride- (Merkord and Hennighausen, 1989) and coxsackievirus- (De Palma et al., 2008, 2009) induced models are also not very widely used due to the lack of major clinical relevance. As for drug-induced AP (Forsmark et al., 2016; Meczker et al., 2020), different responses to drugs between animals and humans make this particularly difficult to model.

## *In vitro* and *Ex vivo* Models

### *In vitro* and *Ex vivo*

Besides animal models, *in vitro* experimental AP models primary PACs, acinar carcinoma cell lines AR42J (rat) (Lugea et al., 2017a) and 266-6 (mouse) (Siveke et al., 2008), and isolated pancreatic lobules (Won et al., 2011) are often used. In 1978, Williams et al. (1978) first developed a technique to isolate PACs and tested the effects of supramaximal doses of CCK and caerulein. Mouse PACs express CCK receptors, of both high and low affinity, which can be activated by low and high concentrations of caerulein/CCK further activating intracellular signaling pathways (summarized in Supplementary Table 1) (Williams, 2001, 2002). Low physiological doses of CCK (or caerulein) bind to high-affinity CCK receptors and mediate pancreatic secretion and growth, whereas high or supra-physiological doses (a concentration exceeding the dose at which maximal amylase secretion is observed) bind to low affinity receptors and inhibit pancreatic secretion, resulting in zymogen activation and PAC injury (Williams, 2001, 2002; Saluja et al., 2007). Grady et al. (1998) and Saluja et al. (1999) reported that no enzyme activation occurred at CCK concentrations below 10^−10^ M, whereas supramaximal stimulation by CCK (concentration >10^−10^ M) results in zymogen activation and PACs injury. Although CCK receptors are abundant in mouse PACs, it is controversial whether CCK receptors exist in human PACs. In 2001, Ji et al. reported that CCK does not cause any changes in human PAC function as was assessed by Ca^2+^ release, amylase secretion, and ERK phosphorylation (Ji et al., 2001). Subsequently, some researchers have reported that CCK can cause Ca^2+^ oscillation and stimulate enzyme secretion in human PACs (Murphy et al., 2008; Wen et al., 2015; Mukherjee et al., 2016; Lugea et al., 2017a,b; Waldron et al., 2019). We have summarized some key information about these studies in Supplementary Table 2.

Subsequent studies carried out in animal models that simulate the human disease suggested that the PACs were the initial site of morphological damage (Lerch et al., 1992). The latest reviews summarizes the effects of CCK on PACs (Williams, 2019) and early acinar events in the pathogenesis of AP (Saluja et al., 2019). The most notable limitation of primary PACs is their *in vitro* viability is relatively short and thus they cannot be used for long-term experiments or subculturing, an advantage that acinar carcinoma cell lines can offer. However, one must be note that while acinar carcinoma cell lines retain some phenotypes of primary PACs (i.e., containing a multitude of digestive enzyme mRNAs and responding to CCK), they may have changed enzyme activities and receptors or start to express other specific receptors.

In contrast to the methods that yield single PACs, isolation of the pancreatic lobules (Won et al., 2011; Gryshchenko et al., 2016) offers a more physiological *ex vivo* model to study signaling events in the exocrine pancreatic tissue. Since the lobules preserve most of the spatial characteristics of the intact acini and contain non-PACs such as such as pancreatic stellate cells (PSCs), they can be used to study the role of interactions between different cell types in the development AP. Lobules of size up to 1 mm^3^ may be manually cut from a saline-injected mouse pancreas (Won et al., 2011), whereas much smaller lobules can be isolated from the organ using a modified protocol of collagenase digestion (Ferdek et al., 2016; Gryshchenko et al., 2016; Jakubowska et al., 2016). Loading the lobules with fluorescent Ca^2+^ indicators has made it possible to investigate how different populations of pancreatic cells orchestrate physiological and pathological Ca^2+^ signals (Won et al., 2011; Ferdek et al., 2016; Gryshchenko et al., 2016; Jakubowska et al., 2016). This model was successfully used to show that Ca^2+^ signal evoked in cells of one phenotype is not transmitted to different cells (Won et al., 2011). Using the multiphoton imaging and Fluo-4 AM Ca^2+^ indicator, Won et al. (2011) confirmed that, even in a close vicinity, different cell populations may form entirely separated signaling units. Gryshchenko et al. (2016) later confirmed that blockade of the bradykinin B2 receptor with antagonist WIN64338 reduces PAC necrosis in the lobules, elicited by common toxins of the pancreas: ethanol, FAEE, or bile acids. Further analyses of the lobular signaling pointed toward more toxic effects of certain bile acids in PSCs than in PACs (Ferdek et al., 2016): this study showed that sodium bile salts, cholate, and taurocholate, elicit noxious Ca^2+^ signals in PSCs, and induce considerable levels of PSC necrosis in the lobules, whereas TLCS mainly induces pathological Ca^2+^ signals and necrotic cell death in PACs. Importantly, development of the real-time method to simultaneously record Ca^2+^ signals (Fura-2 indicator) and nitric oxide signals (DAF-2 fluorescent dye) in the lobules, confirmed the interplay between these signaling pathways solely in PSCs, and their absence in PACs, in response to stimulation of the lobules with bradykinin (Jakubowska et al., 2016). PSCs generated Ca^2+^ responses in form of Ca^2+^ oscillations or transients, accompanied by the intracellular increase in nitric oxide, in response to bradykinin, cholate, and taurocholate, but not TLCS. In a mouse model of alcohol-induced AP, lobular PSCs were desensitized to the signaling mediated by bradykinin B2 receptor, but sensitized to the signaling mediated by B1 receptor (Gryshchenko et al., 2018). This sensitivity switch is a general feature of inflammatory diseases (Petho and Reeh, 2012). The aforementioned studies demonstrate that pancreatic lobules are a valuable alternative to single cell type *in vitro* models, particularly in the early stages of investigation. This model could potentially be developed further to investigate signaling events in pancreatic lobules of human origin.

Normal exocrine functions of the pancreas center around pancreatic juice secretion (Hegyi et al., 2011; Hegyi and Petersen, 2013). In order to form this enzyme-rich alkaline fluid, cells of the acinar compartment cooperate with the epithelia of pancreatic ducts. PACs secrete enzymes capable of hydrolysis of the proteins, polysaccharides, lipids, and nucleic acids, whereas pancreatic ductal cells (PDCs) release isotonic solution rich in bicarbonate that aids the transport of the enzymes into the duodenum and neutralizes the gastric acid (Lee et al., 2012). Not only PACs but also PDCs play their roles in the development of AP. For example, obstruction of the pancreatic duct can influence the trafficking of PAC membrane (Lerch et al., 1995a); and inducers of AP, such as bile acids, reduce the intracellular pH of PDCs (Venglovecz et al., 2008). The technique of PAC isolation via enzymatic digestion of the pancreas has been widely applied in the past few decades to study the physiology and pathophysiology of these cells (Thorn et al., 1993). Similarly, the protocol of pancreatic duct isolation was initially developed in the 80's and it is also based on enzymatic digestion of the tissue (Argent et al., 1986). However, this enzymatic processing could affect the physiology of ductal epithelia and thus may require additional steps facilitating ductal regeneration, which extends and complicates the entire procedure (Gal et al., 2019). In order to address this limitation, Gal et al. recently proposed a novel *in situ* approach that aims to preserve the physiological environment and the ductal structure of the mouse pancreatic tissue (Gal et al., 2019). By injecting (post-mortem) low melting point agarose into the pancreatic duct and a subsequent cooling of the whole organ, agarose settles in the pancreatic ductal system and helps to maintain its three dimensional architecture. Importantly, by using a vibratome, slices of the agarose-reinforced pancreatic tissue can be cut and used for the subsequent analyses, such as measurements of Ca^2+^ signals in real-time (Gal et al., 2019). The authors showed that treatment of PDCs with 1 mM chenodeoxycholic acid evokes robust transient increases in intracellular Ca^2+^ and triggers cell movement that precedes the development of Ca^2+^ responses in these cells (Gal et al., 2019). This new *in situ* model is likely to be a very useful tool for testing the effects of the common inducers of AP on PDCs and may add to our knowledge about the roles these cells play in pancreatic pathologies (Supplementary Table 3).

While models of the isolated mouse pancreatic ducts offer means for studying the functional characteristics of the epithelial surfaces, they are not designed to investigate ducts as structures that form fluid-filled cavities. This potential limitation can be addressed by employing pancreatic ductal organoid cultures (Boj et al., 2015) for analyzing ion secretion (Molnar et al., 2020). Ductal epithelia can be grown as organoids with a hollow center (the lumen), which preserves the planar cell polarity and the physiological pattern of the membrane transporters. In order to measure real time changes in the intraluminal pH, concentration of chloride anions or the processes of bicarbonate secretion, the lumen can be injected with an appropriate fluorescent indicator (Molnar et al., 2020). In the near future, organoid cultures might aid the preclinical studies on the disrupted physiological processes that drive the development of pancreatic disorders.

## Comparison of Commonly Used Models in Rodents

Not all animal models of AP developed in the past are equally popular among researchers today. It is very difficult to compare models of AP due to the differences between animals and strains used, the microbiome and the environmental cues, or reagents/experimental conditions. There is a growing trend toward the use of PDI-AP and PDL-AP models particularly in genetic studies. L-arginine and other amino acids-induced animal models in rodents are now undergoing thorough investigation. Current animal models of AP are classified for severity on the basis of an inducer/etiology that causes pancreatic necrosis (Lerch and Gorelick, 2013). In contrast, the presence of necrosis in the human disease does not automatically translate into poorer outcomes. This is a significant limitation since severity in human AP is unrelated to pancreatic necrosis or etiology, with the exception of hypertriglyceridemia-associated AP. The comparison of currently used models is summarized in Table 4.

**Table 4:** Comparison of commonly used acute pancreatitis animal models in rodents.

**Animal models**	**Clinical relevance**	***In vitro* parallel**	**Severity**	**Mortality**	**Systemic injury**	**Systemic toxicity**	**Invasive**	**Advantages**	**Disadvantages**	**References**
Caerulein (rats/mice)	Scorpion bites or organophosphate insecticides	Yes	Adjustable severity from oedematous to necrotising pancreatitis by number of caerulein (normally 50 μg/kg) injections	No	Yes	No	No	• Injection only required so easy to perform • Large wealth of pre-clinical data exists using model	• Rare clinical parallel	Lampel and Kern, 1977; Niederau et al., 1985; Saluja et al., 2007
L-arginine (rats/mice)	Limited	Yes	Adjustable severity from mild, moderate to severe necrotizing pancreatitis by L-arginine dose, or number of injections	Adjustable mortality	Yes	Yes	No	• Injection only required so easy to perform • Adjustable mortality useful for severity assessment within the same model	• Limited clinical relevance • Potential for systemic toxicity	Tashiro et al., 2001; Hegyi et al., 2004; Dawra et al., 2007
^*^Lieber–DeCarli diet plus other stimuli (rats/mice)	Alcoholic excess	Yes	Moderate to severe pancreatitis	No	Yes	No	No	• Easy to perform • Moderate clinical relevance	• Limited ability to modulate in terms of severity	Pandol et al., 2003; Perides et al., 2005; Lugea et al., 2010
Ethonal/FFA (mice)	Alcoholic excess	Yes	Moderate to severe pancreatitis	Yes	Yes	Yes	No	• Injection only required so easy to perform • Strong clinical relevance	• Potential for Systemic toxicity • Difficult to modulate potential for mortality	Huang et al., 2014, 2017; Wen et al., 2015; Mukherjee et al., 2016
PD perfusion (rats/mice)	Biliary	Yes	Moderate to severe pancreatitis depending on bile acids and their concentrations	Adjustable mortality	Yes	Yes	Yes	• Strong clinical relevance • Adjustable mortality useful for severity assessment within the same model	• Requires micro-surgical training • Potential for systemic toxicity and detergent effects of the bile salts	Schmidt et al., 1992a,b; Laukkarinen et al., 2007; Perides et al., 2010
PD ligation (rats/mice) or CPBD ligation (opossum)	Gallstone obstruction	No	Moderate to severe pancreatitis	Yes	Yes	No	Yes	• Strong clinical relevance • No potential for systemic toxicity	• Requires micro-surgical training	Lerch et al., 1993; Mooren et al., 2003; Meyerholz and Samuel, 2007; Samuel et al., 2010
Post-ERCP pancreatitis (rats/mice)	Post-ERCP pancreatitis	No	Mild to moderate pancreatitis	No	No	No	Yes	• Strong clinical relevance • No potential for systemic toxicity	• Requires micro-surgical training • No systemic injury observed	He et al., 2003; Hackert et al., 2004; Xiong et al., 2007; Noble et al., 2008; Jin et al., 2015; Radadiya et al., 2019

* *Lieber–DeCarli diet is to mix alcohol into a liquid diet (supplemented with calories); FFA: fatty acid acid; PD, pancreatic duct; CPBD, common pancreatic biliary duct; ERCP, endoscopic retrograde cholangiopancreatography*.

## Experimental AP Models in Translational Research

In recent years, there has been an increasing number of medical studies in the pancreatic field (Mukherjee et al., 2019). Animal models play here an indispensable role by bridging basic science with translational research. Animal models allow detailed investigation of the crucial events in the pathophysiology of the disease and are thus important in establishing causality. The names, targets, models used and whether they are effective for system damage caused by AP are summarized in Table 5. However, it pays to remember that even very promising findings made on animal models, particularly those regarding novel therapeutic agents, may not always show efficacy in clinical trials (Moggia et al., 2017). The latter caveat may be improved by understanding critical thresholds for cellular events (Barreto et al., 2020) and assessing potential pharmacological therapeutics in different models including *in vivo* and *in vitro* with multiple biochemical, immunological and histopathological indices, before designing appropriate clinical trials. Some drugs with good curative effect on animal models that successfully transformed into clinical trials are shown in Table 6. It is also crucial to understand that in animal studies the therapeutic agent is most often administered prophylactically i.e., before or simultaneously with the administration of AP. However, in majority of clinical trials treatment can only be instituted after the onset of symptoms. Hence the main focus of animal studies has been toward understanding the mechanistic pathways involved in the pathogenesis of disease. To date, there is no perfect model that shares all typical characteristics of human AP and the failure of anti-inflammatory (Kingsnorth et al., 1995; Kingsnorth, 1996; Johnson et al., 2001), antioxidants (Siriwardena et al., 2007) and antibiotics (Dellinger et al., 2007; Bai et al., 2008; Garcia-Barrasa et al., 2009) to ameliorate AP in human clinical trials, despite their success in animal models, demonstrates well the difficulties in translating the results from animal studies to the clinic. Therefore, there is a long way to go from animal experiment to clinical transformation. Considering these factors it is vital that looking to the future we embrace advances in cellular technologies and in particular human organoids that may provide improved and more representative models (Kim et al., 2020).

**Table 5:** Pharmacological therapy tested in pre-clinical AP models.

**Agent**	**Target**	**Effect**	***in vitro***	**Model**	**Pancreas**	**System**	**References**
Caffeine	IP_3_R	Inhibitor	Mouse PACs	TLCS-AP, CER-AP, FAEE-AP	Yes	No	Huang et al., 2017
GSK-7975A	ORAI1	Inhibitor	Human PACs, Mouse PACs	TLCS-AP, CER-AP, FAEE-AP	Yes	Yes	Wen et al., 2015
CM4620	ORAI1	Inhibitor	Human PACs, Mouse PACs	TLCS-AP, FAEE-AP	Yes	Yes	Wen et al., 2015
DEB025	Cyclophilins	Inhibitor	Human PACs, Mouse PACs	TLCS-AP, CER-AP	Yes	Yes	Mukherjee et al., 2016
TRO40303	Cyclophilins	Inhibitor	Human PACs, Mouse PACs	TLCS-AP, CER-AP	Yes	Yes	Mukherjee et al., 2016
Vitamin K3	Autophagy	Inhibitor	NA	CER-AP	Yes	NA	Chinzei et al., 2011
CYT387	TBK1/IKKε/JAK	Inhibitor	PDACs	NA	Yes	NA	Yang et al., 2016
Trehalose	Autophagy	Enhancer	NA	ARG-AP	Yes	NA	Biczo et al., 2018
CID755673	PKD & NF-κB	Inhibitor	Rat PACs	CER-AP	Yes	NA	Yuan et al., 2017
CRT0066101	PKD & NF-κB	Inhibitor	Rat PACs	CER-AP	Yes	NA	Yuan et al., 2017
IRS954	TLR9	Antagonist	NA	TLCS-AP, CER-AP	Yes	NA	Hoque et al., 2011
Cl-amidine	NETosis	Inhibitor	NA	NaTC-AP	Yes	Yes	Madhi et al., 2019
Chloroquine	NETosis	Inhibitor	NA	ARG-AP, CDE-AP	Yes	Yes	Murthy et al., 2019
Infliximab	TNF-α	Antibody	NA	NaTC-AP	Yes	NA	Tekin et al., 2015
Infliximab	TNF-α	Antibody	Rat peritoneal macrophages	NaTC-AP	Yes	Yes	Luo et al., 2010
Somatostatin	Somatostatin receptor	Agonist	NA	PDL-AP	Yes	NA	Baxter et al., 1985
Octreotide	Somatostatin receptor	Agonist	NA	NaTC-AP	Yes	Yes	Huang et al., 2012
Ulinastatin	Serine protease	Inhibitor	NA	NaTC-AP	Yes	Yes	Pan et al., 2017
GSK180	Kynurenine-3-monooxygenase	Inhibitor	NA	NaTC & CER-AP	Yes	Yes	Mole et al., 2016

*NA, not available; IP3R, inositol trisphosphate receptor; ORAI1, calcium release-activated calcium modulator 1; TBK1, TANK-binding kinase 1; IKKε, inhibitor of kappa B kinase epsilon; JAK, Janus kinase; PKD, protein kinase D; NF-κB, nuclear factor kappa-B; TLR9, Toll-like receptor 9; TNF-α, tumor necrosis factor alpha; PACs, pancreatic acinar cells; PDAC, pancreatic ductal adenocarcinoma; CER-AP, caerulein-induced acute pancreatitis; CDE-AP, CDE-diet induced acute pancreatitis; NaTC-AP, sodium taurocholate-induced acute pancreatitis; ARG-AP, L-arginine-induced acute pancreatitis; TLCS-AP, taurolithocholic acid 3-sulfate induced acute pancreatitis; PDL-AP, pancreatic duct ligation-induced acute pancreatitis; FAEE-AP, fatty acid ethyl ester-induced acute pancreatitis*.

**Table 6:** Pharmacological therapy for AP in currently clinical trials.

**Agent**	**Conditions**	**Status**	**Number**	**Site**	**Locations**	**Phase**	**NCT Number**
CM4620	Acute pancreatitis	Recruiting	42	Single	United States	Phase 1; Phase 2	NCT04195347
	Acute pancreatitis; SIRS	Completed	21	Multiple	United States	Phase 2	NCT03401190
Infliximab	Acute pancreatitis	Recruiting	290	Single	United Kingdom	Phase 2	NCT03684278
Somatostatin	Post-ERCP pancreatitis	Completed	300	Single	Greece	NA	NCT00222092
Octreotide	Post-ERCP pancreatitis	Completed	300	Single	Greece	NA	NCT00222092
Ulinastatin	Acute pancreatitis	Suspended	252	Multiple	China	Phase 4	NCT01132521
CytoSorb	Acute pancreatitis; SIRS	Unknown	30	NA	NA	Phase 4	NCT03082469

*NA, not available; SIRS, systemic inflammatory response syndrome; ERCP, endoscopic retrograde cholangiopancreatography*.

## Summary

Animal models of AP, whether they are invasive or non-invasive, carried out on large or small animals, wild type or transgenic animals, have provided and continue to provide key insights into the etiology and pathogenesis of AP as well as aid the identification of new therapeutic targets or biomarkers useful for the treatment of the disease. They remain an indispensable tool for the study of AP. As our understanding of the disease continues to improve, it is likely that new and more relevant models will be developed in the near future.

## Data Availability Statement

The raw data supporting the conclusions of this article will be made available by the authors, without undue reservation.

## Author Contributions

XY and LY performed literature search and drafted the manuscript. PF, MJ, and RM reviewed and critically revised the manuscript. XF and QX obtained funding and had important intelligent input. WH obtained funding, conceptualized the study, supervised students, drafted, and critically revised the manuscript. All authors have read and agreed to the published version of the manuscript.

## Conflict of Interest

The authors declare that the research was conducted in the absence of any commercial or financial relationships that could be construed as a potential conflict of interest.
