# Supplementary material for: Experimental Acute Pancreatitis Models: History, Current Status, and Role in Translational Research
Source: Front Physiol. 2020 Dec 23;11:614591. doi: 10.3389/fphys.2020.614591 (PMC7786374; doi:10.3389/fphys.2020.614591)
Supplement: Supplementary file 1 [file Table_1.DOCX]

Supplementary Table

**Supplementary Table 1. Secretagogue**

| **Secretagogue** | **Concentration** | **Cell** | **Effects** | **References** |
| --- | --- | --- | --- | --- |
| CCK | 100 nM | PACs | Loss of ΔΨm, reduction of ATP levels | (Shen et al., 2018) (Biczo et al., 2018) |
|  | 100 nM | PACs | Degradation of long-lived proteins, a measure of autophagic efficiency, was markedly inhibited | (Mareninova et al., 2009) |
|  | 10 pM | PACs | A rapid (within 1 minute) decrease in Δψm followed by a plateau sustained for up to 16 minutes, and then Δψm recovered back to the basal level, stimulated intracellular Ca^2+^ oscillations | (Shalbueva et al., 2013) |
|  | 0.1 nM, 100 nM | PACs | A decrease in protein degradation, concomitant with accumulation of autophagic vacuoles | (Mareninova et al., 2009) |
|  | 0.001-100 nM | PACs | CCK stimulation induced a typical biphasic dose response curve for amylase secretion in acinar cells with maximal stimulation at 10 pM CCK. CCK (100 nM) induced zymogen and NF-κB activation. | (Thrower et al., 2009) |
|  | 10^-10^-10^-8^ M | PACs | 10^-10^ M CCK induces the maximal secretory response, whereas 10^-8^ M CCK represents a supramaximal concentration leading to inhibition of secretion. 10^-8^ M CCK stimulation causes plectin breakdown and activation of caspase 8. Enzymatic activities reached their maximum at 10^-8^ M CCK | (Lampel and Kern, 1977) |
|  | 10^-11^, 10^-9^, 10^-7^ M | PACs | CCK stimulates activity of caspases-3, -8, and -9. CCK stimulates Caspase-dependent cleavage of protein kinase Cδ. CCK induces apoptosis in pancreatic acinar cells that is mediated by caspases. CCK dose-dependently stimulates cytochrome c release and mitochondrial depolarization; the former but not the latter, is prevented by PTP inhibitors | (Gukovskaya et al., 2002) |
|  | 100 nM | PACs | CCK activated the novel isoforms PKC-δ and -∈ and the atypical isoform PKC-ξ but not the conventional isoform PKC-α. Inhibition of the novel PKC isoforms but not the conventional or the atypical isoform resulted in the prevention of NF-κB activation induced by CCK. | (Dressel et al., 1982) |
|  | 0.1 nM, 100 nM | PACs | 100 nM CCK caused both NF-κB and PKC-δ, -∈, and -ξ activation, whereas 0.1 nM CCK did not increase PKC-∈, PKC-ξ, or NF-κB activity. | (Satoh et al., 2006) |
|  | 10 nM | PACs | CCK (10 nM) induced large cytosolic calcium elevations, falls in Δψm and NAD(P)H autofluorescence, increased trypsin activity | (Mukherjee et al., 2016) |
|  | 10 pM, 100 pM | PACs | CCK (10 pm) increases secretion of amylase and lipase by ~2-fold over the basal value at control and the release of FFAs . Release of LDH is not significantly increased after 30 min of stimulation with CCK up to a CCK concentration of 100 pM | (Wang et al., 2008; Yang et al., 2009) |
|  | 2, 5, 10 pM | PACs | ΔΨm not responded to 2 pM eventually responded to either 5 or 10 pM CCK. 20 or 50 nM loss in Δψm | (Voronina et al., 2004) |
| Caerulein | 10 nM | PACs | Cell damage (trypan blue & LDH) | (Pallagi et al., 2014) |
|  | 100 nM | PACs | 100 nM caerulein stimulation caused PKC-δ and -ε isoform translocation to microsomal membranes | (Thrower et al., 2008) |
|  | 100 nM | PACs | Causes NF-κB activation | (Muili et al., 2013) |
|  | 100 nM | PACs | Increase amylase secretion | (Sonda et al., 2013) |
|  | 100 nM | PACs | Low pH enhances cerulein-induced zymogen activation over a range of cerulein concentrations (0.01 nM-100 nM) without significantly affecting amylase secretion. The effects are reversible. The vATPase inhibitor concanamycin blocks cerulein-induced zymogen activation over a range of pHs. Low pH enhances cerulein-induced cell injury (trypan blue & LDH). | (Bhoomagoud et al., 2009) |
|  | 100 nM | PACs | Increases Necrotic cell death (both PI uptake and LDH leakage), didn’t trigger either TUNEL staining or an increase in caspase activity. ATP depletion, Accelerated calcium influx and pathologic increases of cytoplasmic calcium levels | (Louhimo et al., 2016) |
|  | 0.01, 0.1, 1, 10 nM | PACs | A typical biphasic dose-response with basal amylase release, trypsinogen activation, cathepsin B activation and cell death | (Cendrowski et al., 2015) |
|  | 10^-12^ M to 10^-7^ M | PACs | Increase IL-6 expression and NF-κB activity, increase in amylase release | (Bhatia et al., 2014) |
|  | 10 pM | PACs | Physiological calcium transients characterized by an oscillatory response | (Perides et al., 2010) |
|  | 10 nM | PACs | Pathological calcium transients, digestive zymogen activation, concentration-dependent increases in amylase discharge | (Perides et al., 2010) |
| Caerulein & IL-6 | Caerulein 100 nM & IL-6 10^-6^ g/L | AR42J | Apoptosis and necrosis (CCK), ROS, increased the expression of phosphor-NF-κB p65 and caspase-1 | (Banks et al., 2006) |

**NOTE.** PACs, pancreatic acinar cells; ATP, adenosine triphosphate; Δψm, mitochondrial membrane potential; CCK, cholecystokinin; NF-κB, nuclear factor kappa-B; PTP, permeability transition pore; FFAs, free fatty acids; PI, propidium iodide; LDH, lactate dehydrogenase; PKC, protein kinase C; ROS: reactive oxygen species; IL-6, interleukin 6.

**Supplementary Table 2. Human acinar cells**

| **Year** | **Time** | **Medium** | **trypsin inhibitor** | **PH** | **Inducer** | **Findings** | **References** |
| --- | --- | --- | --- | --- | --- | --- | --- |
| 2001 | < 1 hour | HEPES | Yes | NA | CCK; gastrin; CCh | Did not respond to CCK agonists; but responded to carbachol; insufficient level of receptor expression | (Ji et al., 2001) |
| 2008 | < 10 mins | HEPES | Yes | 7.35 | CCK; human  CCK-58 | Ca^2+^ oscillation; activates mitochondrial function, and stimulates enzyme secretion | (Murphy et al., 2008) |
| 2015 | < 10 mins | HEPES | Yes | 7.35 | Thapsigargin;  TLCS | PI uptake; Ca^2+^ influx | (Wen et al., 2015) |
| 2016 | < 10 mins | HEPES | Yes | 7.35 | TLCS | Induced MPTP opening; decreases of Δψm; increases PI uptake | (Mukherjee et al., 2016) |
| 2017 | < 12 hours | Media 199 | Yes | 7.4 | CCh; TLCS | Expressed the muscarinic acetylcholine receptor M3 and maintained physiological responses to CCh for at least 20 hours; induced trypsinogen activation, decreased cell viability, organelle damage manifest by mitochondrial depolarization, disordered autophagy, and pathological endoplasmic reticulum stress; secreted inflammatory mediators including IL-6, TNF-α, IL-1β, chemokine (C-C motif) ligands 2 and 3, macrophage inhibitory factor, and chemokines mediating neutrophil and monocyte infiltration | (Lugea et al., 2017b) |
| 2017 | NA | Media 199 | Yes | 7.4 | EtOH; CSE | Increases PI uptake | (Lugea et al., 2017a) |
| 2019 | NA | Media 199 | Yes | 7.4 | CCh; TLCS | Increases PI uptake; increases trypsin activity | (Waldron et al., 2019) |

**NOTE.** CCK, cholecystokinin; CCh, carbachol; CSE, cigarette smoke extract; EtOH, ethanol; PI, propidium iodide; TLCS, taurolithocholic acid 3-sulphate; Δψm, mitochondrial membrane potential; IL-6, interleukin 6; TNF-α, tumor necrosis factor alpha; IL-1β, interleukin-1 beta.

Supplementary Table 3. Pancreatic ductal epithelial cells.

| **Drugs** | **Concentration** | **Cell** | **Effects** | **Reference** |
| --- | --- | --- | --- | --- |
| CDC | 1 mM | PDECs | Strong inhibitory effects on the activities of acid/base transporters (Na^+^/H^+^ exchanger (NHE), Na^+^/HCO^3-^ cotransporter (NBC) and Cl^-^/HCO^3-^ exchanger (CBE)) damaged all of the mitochondria, other intracellular organelles such as nuclei or Golgi apparatus seemed to be unaltered. Decreased ATP_i_, inhibits pancreatic ductal bicarbonate secretion | (Saluja et al., 1999) |
|  | 1 mM | PDECs | Strongly inhibits ductal HCO^3-^ secretion | (Venglovecz et al., 2011) |
|  | 0.1 mM | PDECs | No effects on the intracellular organelles | (Saluja et al., 1999) |
|  | 100 μM | PDECs | Increased whole cell K+ currents and hyperpolarised cell membrane potential. stimulates HCO^3-^ secretion via elevation of intracellular Ca^2+^. | (Venglovecz et al., 2011) |
|  | 0.1 mM | PDECs | 0.1 mM stimulated ductal HCO^3-^ secretion, 1 mM strongly inhibited HCO^3-^secretion, dose-dependent increase in intracellular Ca^2+^ | (Venglovecz et al., 2008) |
| CDCA | 1 mM | PDECs | ATP depletion, mitochondrial injury, and cell death (kit), Ca^2+^ signaling, induced a dose-dependent intracellular acidification in HEPES buffered solution | (Katona et al., 2016) |
| UDCA | 0.5 mM | PDECs | UDCA induced a dose-dependent decrease in pHi, which was much smaller than CDCA. UDCA pretreatment reduced the rate of ATP depletion, mitochondrial injury, and cell death (kit) induced by 1 mM CDCA, prevents the inhibitory effect of CDCA on acid-base transporters, but had no effect on CDCA-induced Ca^2+^ signaling | (Katona et al., 2016) |
| GCDC | 0.1 mM | PDECs | No effects on the intracellular organelles | (Saluja et al., 1999) |
|  | 1 mM | PDECs | Did not induce morphological changes, decreased ATP_i_, dose-dependent increase in intracellular Ca^2+^ | (Saluja et al., 1999; Venglovecz et al., 2008) |
| Ethanol  Ethanol | 1, 10, 100 mM | PDECs | 10 mM significantly increased the basal current, 100 mM ethanol induced a robust increase within 5 min,100 mM cause ATP_i_ depletion | (Judak et al., 2014) |
|  | 100 mM | PDECs | 30 minutes markedly reduced pancreatic fluid secretion, significantly diminished ductal HCO^3−^ secretion | (Maleth et al., 2015) |
|  | 100 mM | Capan-1 | 15-minute administration of a low concentration of ethanol (10 mM) stimulated whereas a high concentration of ethanol (100 mM) and POA (100, 200 μM) significantly impaired the apical CI^−^ /HCO^3−^ exchange activity. 100 mM ethanol and 100 to 200 μM POA significantly inhibited the recovery from acid load during NH_4_Cl pulse experiments under basal conditions and forskolin stimulation. | (Maleth et al., 2015) |
|  | 0.1–30 mM | PDECs | 0.3–30 mM significantly augmented fluid secretion stimulated by physiological (1 pM) or pharmacological (1 nM) concentrations of secretin. 1 mM shifted the secretin concentration–fluid secretion response curve upwards. In secretin-stimulated ducts, 1 mM ethanol induced a transient increase in intracellular Ca^2+^（low concentrations of ethanol directly augment pancreatic ductal fluid secretion stimulated by physiological and pharmacological concentrations of secretin, and this appears to be mediated by the activation of both the intracellular cAMP pathway and Ca^2+^ mobilization.） | (Yamamoto et al., 2003) |
| Acetaldehyde | 1, 5 mM | PDECs | 1 nor 5 mM Ac had any effect on basal or forskolin-stimulated CFTR currents | (Judak et al., 2014) |
| POAEE | 10-200 μM | PDECs | 10–200 μM had no effect on the basal currents, 200 mM caused ATP_i_ depletion | (Judak et al., 2014) |
|  | 200 μM | PDECs | No effect on pancreatic fluid secretion | (Maleth et al., 2015) |
| POA | 10, 100, 200 μM | PDECs | 10 μM no affect the basal, 100 and 200 μM POA induced a dose-dependent and significant decrease in both the basal currents, 200 mM cause ATP_i_ depletion | (Judak et al., 2014) |
|  | 200 μM | PDECs | 30 minutes markedly reduced pancreatic fluid secretion, significantly diminished ductal HCO^3−^ secretion | (Maleth et al., 2015) |
| Mannitol | 177 mM | PDECs | Increase in whole cell currents | (Judak et al., 2014) |

**NOTE.** CDC, chenodeoxycholate; GCDC, glycochenodeoxycholate; CDCA, chenodeoxycholic acid; UDCA, ursodeoxycholic acid; PDECs, pancreatic ductal epithelial cells.

**Reference**

Banks, P.A., Freeman, M.L., and Practice Parameters Committee of the American College of, G. (2006). Practice guidelines in acute pancreatitis. Am J Gastroenterol *101*, 2379-2400.

Bhatia, V., Rastellini, C., Han, S., Aronson, J.F., Greeley, G.H., Jr., and Falzon, M. (2014). Acinar cell-specific knockout of the PTHrP gene decreases the proinflammatory and profibrotic responses in pancreatitis. Am J Physiol Gastrointest Liver Physiol *307*, G533-549.

Bhoomagoud, M., Jung, T., Atladottir, J., Kolodecik, T.R., Shugrue, C., Chaudhuri, A., Thrower, E.C., and Gorelick, F.S. (2009). Reducing extracellular pH sensitizes the acinar cell to secretagogue-induced pancreatitis responses in rats. Gastroenterology *137*, 1083-1092.

Biczo, G., Vegh, E.T., Shalbueva, N., Mareninova, O.A., Elperin, J., Lotshaw, E., Gretler, S., Lugea, A., Malla, S.R., Dawson, D.*, et al.* (2018). Mitochondrial Dysfunction, Through Impaired Autophagy, Leads to Endoplasmic Reticulum Stress, Deregulated Lipid Metabolism, and Pancreatitis in Animal Models. Gastroenterology *154*, 689-703.

Cendrowski, J., Lobo, V.J., Sendler, M., Salas, A., Kuhn, J.P., Molero, X., Fukunaga, R., Mayerle, J., Lerch, M.M., and Real, F.X. (2015). Mnk1 is a novel acinar cell-specific kinase required for exocrine pancreatic secretion and response to pancreatitis in mice. Gut *64*, 937-947.

Dressel, T.D., Goodale, R.L., Jr., Zweber, B., and Borner, J.W. (1982). The effect of atropine and duct decompression on the evolution of Diazinon-induced acute canine pancreatitis. Ann Surg *195*, 424-434.

Gukovskaya, A.S., Gukovsky, I., Jung, Y., Mouria, M., and Pandol, S.J. (2002). Cholecystokinin induces caspase activation and mitochondrial dysfunction in pancreatic acinar cells. Roles in cell injury processes of pancreatitis. The Journal of biological chemistry *277*, 22595-22604.

Ji, B., Bi, Y., Simeone, D., Mortensen, R.M., and Logsdon, C.D. (2001). Human pancreatic acinar cells lack functional responses to cholecystokinin and gastrin. Gastroenterology *121*, 1380-1390.

Judak, L., Hegyi, P., Rakonczay, Z., Jr., Maleth, J., Gray, M.A., and Venglovecz, V. (2014). Ethanol and its non-oxidative metabolites profoundly inhibit CFTR function in pancreatic epithelial cells which is prevented by ATP supplementation. Pflugers Arch *466*, 549-562.

Katona, M., Hegyi, P., Kui, B., Balla, Z., Rakonczay, Z., Jr., Razga, Z., Tiszlavicz, L., Maleth, J., and Venglovecz, V. (2016). A novel, protective role of ursodeoxycholate in bile-induced pancreatic ductal injury. Am J Physiol Gastrointest Liver Physiol *310*, G193-204.

Lampel, M., and Kern, H.F. (1977). Acute interstitial pancreatitis in the rat induced by excessive doses of a pancreatic secretagogue. Virchows Arch A Pathol Anat Histol *373*, 97-117.

Louhimo, J., Steer, M.L., and Perides, G. (2016). Necroptosis Is an Important Severity Determinant and Potential Therapeutic Target in Experimental Severe Pancreatitis. Cell Mol Gastroenterol Hepatol *2*, 519-535.

Lugea, A., Gerloff, A., Su, H.Y., Xu, Z., Go, A., Hu, C., French, S.W., Wilson, J.S., Apte, M.V., Waldron, R.T.*, et al.* (2017a). The Combination of Alcohol and Cigarette Smoke Induces Endoplasmic Reticulum Stress and Cell Death in Pancreatic Acinar Cells. Gastroenterology *153*, 1674-1686.

Lugea, A., Waldron, R.T., Mareninova, O.A., Shalbueva, N., Deng, N., Su, H.Y., Thomas, D.D., Jones, E.K., Messenger, S.W., Yang, J.*, et al.* (2017b). Human Pancreatic Acinar Cells: Proteomic Characterization, Physiologic Responses, and Organellar Disorders in ex Vivo Pancreatitis. Am J Pathol *187*, 2726-2743.

Maleth, J., Balazs, A., Pallagi, P., Balla, Z., Kui, B., Katona, M., Judak, L., Nemeth, I., Kemeny, L.V., Rakonczay, Z., Jr.*, et al.* (2015). Alcohol disrupts levels and function of the cystic fibrosis transmembrane conductance regulator to promote development of pancreatitis. Gastroenterology *148*, 427-439 e416.

Mareninova, O.A., Hermann, K., French, S.W., O'Konski, M.S., Pandol, S.J., Webster, P., Erickson, A.H., Katunuma, N., Gorelick, F.S., Gukovsky, I.*, et al.* (2009). Impaired autophagic flux mediates acinar cell vacuole formation and trypsinogen activation in rodent models of acute pancreatitis. J Clin Invest *119*, 3340-3355.

Muili, K.A., Jin, S., Orabi, A.I., Eisses, J.F., Javed, T.A., Le, T., Bottino, R., Jayaraman, T., and Husain, S.Z. (2013). Pancreatic acinar cell nuclear factor kappaB activation because of bile acid exposure is dependent on calcineurin. J Biol Chem *288*, 21065-21073.

Mukherjee, R., Mareninova, O.A., Odinokova, I.V., Huang, W., Murphy, J., Chvanov, M., Javed, M.A., Wen, L., Booth, D.M., Cane, M.C.*, et al.* (2016). Mechanism of mitochondrial permeability transition pore induction and damage in the pancreas: inhibition prevents acute pancreatitis by protecting production of ATP. Gut *65*, 1333-1346.

Murphy, J.A., Criddle, D.N., Sherwood, M., Chvanov, M., Mukherjee, R., McLaughlin, E., Booth, D., Gerasimenko, J.V., Raraty, M.G., Ghaneh, P.*, et al.* (2008). Direct activation of cytosolic Ca2+ signaling and enzyme secretion by cholecystokinin in human pancreatic acinar cells. Gastroenterology *135*, 632-641.

Pallagi, P., Balla, Z., Singh, A.K., Dosa, S., Ivanyi, B., Kukor, Z., Toth, A., Riederer, B., Liu, Y., Engelhardt, R.*, et al.* (2014). The role of pancreatic ductal secretion in protection against acute pancreatitis in mice*. Crit Care Med *42*, e177-188.

Perides, G., Laukkarinen, J.M., Vassileva, G., and Steer, M.L. (2010). Biliary acute pancreatitis in mice is mediated by the G-protein-coupled cell surface bile acid receptor Gpbar1. Gastroenterology *138*, 715-725.

Saluja, A.K., Bhagat, L., Lee, H.S., Bhatia, M., Frossard, J.L., and Steer, M.L. (1999). Secretagogue-induced digestive enzyme activation and cell injury in rat pancreatic acini. Am J Physiol *276*, G835-842.

Satoh, A., Gukovskaya, A.S., Reeve, J.R., Jr., Shimosegawa, T., and Pandol, S.J. (2006). Ethanol sensitizes NF-kappaB activation in pancreatic acinar cells through effects on protein kinase C-epsilon. Am J Physiol Gastrointest Liver Physiol *291*, G432-438.

Shalbueva, N., Mareninova, O.A., Gerloff, A., Yuan, J., Waldron, R.T., Pandol, S.J., and Gukovskaya, A.S. (2013). Effects of oxidative alcohol metabolism on the mitochondrial permeability transition pore and necrosis in a mouse model of alcoholic pancreatitis. Gastroenterology *144*, 437-446 e436.

Shen, Y., Wen, L., Zhang, R., Wei, Z., Shi, N., Xiong, Q., Xia, Q., Xing, Z., Zeng, Z., Niu, H.*, et al.* (2018). Dihydrodiosgenin protects against experimental acute pancreatitis and associated lung injury through mitochondrial protection and PI3Kgamma/Akt inhibition. Br J Pharmacol *175*, 1621-1636.

Sonda, S., Silva, A.B., Grabliauskaite, K., Saponara, E., Weber, A., Jang, J.H., Zullig, R.A., Bain, M., Reding Graf, T., Hehl, A.B.*, et al.* (2013). Serotonin regulates amylase secretion and acinar cell damage during murine pancreatitis. Gut *62*, 890-898.

Thrower, E.C., Osgood, S., Shugrue, C.A., Kolodecik, T.R., Chaudhuri, A.M., Reeve, J.R., Jr., Pandol, S.J., and Gorelick, F.S. (2008). The novel protein kinase C isoforms -delta and -epsilon modulate caerulein-induced zymogen activation in pancreatic acinar cells. Am J Physiol Gastrointest Liver Physiol *294*, G1344-1353.

Thrower, E.C., Wang, J., Cheriyan, S., Lugea, A., Kolodecik, T.R., Yuan, J., Reeve, J.R., Jr., Gorelick, F.S., and Pandol, S.J. (2009). Protein kinase C delta-mediated processes in cholecystokinin-8-stimulated pancreatic acini. Pancreas *38*, 930-935.

Venglovecz, V., Hegyi, P., Rakonczay, Z., Jr., Tiszlavicz, L., Nardi, A., Grunnet, M., and Gray, M.A. (2011). Pathophysiological relevance of apical large-conductance Ca(2)+-activated potassium channels in pancreatic duct epithelial cells. Gut *60*, 361-369.

Venglovecz, V., Rakonczay, Z., Jr., Ozsvari, B., Takacs, T., Lonovics, J., Varro, A., Gray, M.A., Argent, B.E., and Hegyi, P. (2008). Effects of bile acids on pancreatic ductal bicarbonate secretion in guinea pig. Gut *57*, 1102-1112.

Voronina, S.G., Barrow, S.L., Gerasimenko, O.V., Petersen, O.H., and Tepikin, A.V. (2004). Effects of secretagogues and bile acids on mitochondrial membrane potential of pancreatic acinar cells: comparison of different modes of evaluating DeltaPsim. J Biol Chem *279*, 27327-27338.

Waldron, R.T., Chen, Y., Pham, H., Go, A., Su, H.Y., Hu, C., Wen, L., Husain, S.Z., Sugar, C.A., Roos, J.*, et al.* (2019). The Orai Ca(2+) channel inhibitor CM4620 targets both parenchymal and immune cells to reduce inflammation in experimental acute pancreatitis. J Physiol *597*, 3085-3105.

Wang, Y., Sternfeld, L., Yang, F., Rodriguez, J.A., Ross, C., Hayden, M.R., Carriere, F., Liu, G., Hofer, W., and Schulz, I. (2008). Enhanced susceptibility to pancreatitis in severe hypertriglyceridaemic lipoprotein lipase-deficient mice and agonist-like function of pancreatic lipase in pancreatic cells. Gut *58*, 422-430.

Wen, L., Voronina, S., Javed, M.A., Awais, M., Szatmary, P., Latawiec, D., Chvanov, M., Collier, D., Huang, W., Barrett, J.*, et al.* (2015). Inhibitors of ORAI1 Prevent Cytosolic Calcium-Associated Injury of Human Pancreatic Acinar Cells and Acute Pancreatitis in 3 Mouse Models. Gastroenterology *149*, 481-492 e487.

Yamamoto, A., Ishiguro, H., Ko, S.B., Suzuki, A., Wang, Y., Hamada, H., Mizuno, N., Kitagawa, M., Hayakawa, T., and Naruse, S. (2003). Ethanol induces fluid hypersecretion from guinea-pig pancreatic duct cells. J Physiol *551*, 917-926.

Yang, F., Wang, Y., Sternfeld, L., Rodriguez, J.A., Ross, C., Hayden, M.R., Carriere, F., Liu, G., and Schulz, I. (2009). The role of free fatty acids, pancreatic lipase and Ca+ signalling in injury of isolated acinar cells and pancreatitis model in lipoprotein lipase-deficient mice. Acta physiologica (Oxford, England) *195*, 13-28.
